# SPOTting Model Parameters Using a Ready-Made Python Package

**DOI:** 10.1371/journal.pone.0145180

**Published:** 2015-12-17

**Authors:** Tobias Houska, Philipp Kraft, Alejandro Chamorro-Chavez, Lutz Breuer

**Affiliations:** 1 Institute for Landscape Ecology and Resources Management, Research Centre for BioSystems, Land Use and Nutrition (IFZ), Justus Liebig University, Giessen, Germany; 2 Centre for International Development and Environmental Research, Justus Liebig University, Giessen, Germany; Tennessee State University, UNITED STATES

## Abstract

The choice for specific parameter estimation methods is often more dependent on its availability than its performance. We developed SPOTPY (Statistical Parameter Optimization Tool), an open source python package containing a comprehensive set of methods typically used to calibrate, analyze and optimize parameters for a wide range of ecological models. SPOTPY currently contains eight widely used algorithms, 11 objective functions, and can sample from eight parameter distributions. SPOTPY has a model-independent structure and can be run in parallel from the workstation to large computation clusters using the Message Passing Interface (MPI). We tested SPOTPY in five different case studies to parameterize the Rosenbrock, Griewank and Ackley functions, a one-dimensional physically based soil moisture routine, where we searched for parameters of the van Genuchten-Mualem function and a calibration of a biogeochemistry model with different objective functions. The case studies reveal that the implemented SPOTPY methods can be used for any model with just a minimal amount of code for maximal power of parameter optimization. They further show the benefit of having one package at hand that includes number of well performing parameter search methods, since not every case study can be solved sufficiently with every algorithm or every objective function.

## Introduction

Ecological models are often very complex and contain many parameters that need to be optimized prior to model application. Reliable parameter estimation is highly dependent on various criteria, including the selected algorithm, the objective function and the definition of the prior parameter distribution. Difficulties involved in calibrating for example hydrological models have been partly attributed to the lack of robust optimization tools [1]. Numerous parameterization methods have been developed in the past (e.g. [2–6]), often published without access to the source code. They are widely accepted to determine the values of non-measureable parameters for a model [7]. Many of the methods have been established as part of the parameterization problem in hydrological modeling as early as in the 1990s [8,9]. The application of these methods has now become more widespread in other ecological disciplines and therefore, the methods proposed here, are in fact applicable to a large variety of models in ecology and beyond.

The main goal of parameter optimization is to find one or more sets of parameters, which enables a model to simulate an output with a quasi-optimal objective function. There have been extensive discussions about the best way of model parameterization and calibration [10–12], including dispute about whether there is one optimal parameter set or whether there are several parameter sets of equal behavior (equifinality, [13]). The same is true for the discussion of the best likelihood function to be used [14], how it is determined [15] and the parameter distribution from which parameters should be sampled [16]. Furthermore, improper application of calibration methods can result in misleading parameter estimations [17]. However, nearly no guidance exists which parameter estimation method should be used under specific optimization problems [18]. We want to contribute to these open questions by providing a package that allows investigation of various aspects in model calibration, parameterization and uncertainty analyses. The goal is to help users in testing and finding an efficient technique for their specific parameter search problem.

Numerous ad hoc solutions for the combination of a single calibration/uncertainty method and a single model exist. If one is interested in testing different methods, every solution has to be searched, understood and adopted. This is why in recent years packages where published, providing multiple methods for multiple models. Important ones are: Parameter ESTimation and uncertainty analysis (PEST) [19], the Monte Carlo Analysis Toolbox (MCAT), a parameter estimation toolbox [20] for the Soil Water Assessment Tool (SWAT), OpenBUGS (Bayesian inference Using Gibbs Sampling) [21], STAN [22] and PYMC [23]. However, most of these packages only allow usage of two or three multiple stochastic probabilistic methods. Packages like STAN and PYMC concentrate on Markov Chain Monte Carlo (MCMC) methods. PEST bridges the gap to evolutionary computation methods, a second group of probabilistic global optimization methods [24], like e.g. Shuffled Complex Evolution (SCE-UA) [1], but has no possibility to use e.g. the Generalized Likelihood Uncertainty Estimation method (GLUE) [10,25], which is widely used to address the equifinality problem. MCAT helps to use the GLUE methodology for models. None of these packages covers the wide range of available parameter search methods. Further, no common criteria exist that place the development of such packages in a formal framework. We therefore define five criteria, inspired by the criteria for modern hydrological models [26], which we think are important:

1. Broadness: The available parameter estimation methods should cover a broad range of method families, ranging from path-oriented optimizations to global parameter behavioral uncertainty assessments. This is even more important as no single parameter estimation technique is perfect [27,28] and just very small guidance exists, which parameter estimation approach should be used under specific circumstances [18].
2. Modularity: Parameter estimation packages consist of several modules: the parameter search algorithm, the objective function, a module to save the results of the model runs to disk and the used model. By using a strict modular approach, any given search algorithm can easily be combined with any objective function, giving the user the maximum freedom to adopt a method to a given scientific question.
3. Independency: A model independent package facilitates widespread applications. While a method that is bound to a given model can be used to explore parameter uncertainty, structural model uncertainty remains unquestioned. A model independent method allows the comparison of different model structures using the same parameter space exploration technique and hence the comparison of model structural errors.
4. Scalability: This requirement is an extension of the portability claim [26]. While we agree, that published codes should run both on Windows PC for method testing, as well as on Linux based high performance computing (HPC) systems, scalability goes beyond the portability claim. Scalability means on the one hand, a simple parallelization of the parameter search, where the algorithm allows parallel computation. A package should allow using the parallel power of HPC systems without the need for extensive knowledge of parallel systems. On the other hand, scalability means the possibility to optimize the computational performance of the model. The runtime of models that are fast to evaluate, like e.g. HBV [29] is often dominated by the time needed to load the parameters and input data from disk, and not by the CPU time. Tweaking the model to accept input data through memory can speed up the model evaluation by a magnitude. A scalable package should therefore allow such optimizations and not rely on input file manipulation as an interface between the parameter estimation method and the model alone, as it is the case for most model independent estimation packages.
5. Accessibility: Since a broad, modular package for parameter-estimation carries already a generalized infrastructure for parameter estimation, publishing the package as a free software enables method developers to extend it, without the need to reinvent for example likelihood definitions or parallelization structures. As such, new methods using the existing infrastructure can easily use all existing methods without further development. However, making the source code available for the public is not sufficient for accessibility. The source must also be modular in its structure and well documented, to simplify the adoption of the underlying infrastructure.

We have developed the parameter-spotting package SPOTPY in agreement with these five criteria. We have implemented and tested a wide range of commonly used algorithms into SPOTPY, to allow a user-friendly access to these powerful techniques, and to give an overview, which algorithms and which objective functions can be useful under specific parameter search problems.

## Methods

### Concept of SPOTPY

SPOTPY is broad as it comes along with different global optimization approaches. We included the Monte Carlo (MC), Latin Hyper Cube Sampler (LHS) [3] and Robust Parameter Estimation (ROPE) [5] methods that belong to the first group of stochastic probabilistic methods. They are all-around algorithms, applicable for uncertainty and calibration analysis. MC and LHS can furthermore be utilized within the GLUE methodology. Simulated Annealing (SA) is a heuristic subgroup of the stochastic probabilistic methods. We included a version by Kirckpatrick et al. [6]. The Maximum Likelihood Estimation method (MLE) belongs to the subgroup of hill climbing algorithms and is suited for monotonic response surfaces. Markov Chain Monte Carlo (MCMC) methods, a subgroup of the probabilistic methods, support the ability to jump away from local minima. We implemented the standard Metropolis MCMC sampler [4]. To cover the second group of probabilistic methods (evolutionary algorithms) we included the evolution strategy of SCE-UA. It is suited to calibrate models with high parameter space. Furthermore, the Differential Evolution Markov Chain (DE-MC_Z_) was included to provide a Bayesian solution suited for optimization problems in high parameter space.

SPOTPY is modular since prior parameter distributions, model inputs, evaluation data and objective functions can be selected and combined by the user. The user-defined combination of the inputs can be run with the parameter search algorithms and results are saved either on the working storage or in a csv file. The database structure enables the analyses of the results in SPOTPY with pre-build plotting functions and statistical analyses like Gelman-Rubin diagnostic [30] or the Geweke test [31]. The database can also be used for any other external statistical software or computer language.

SPOTPY is independent as the model is wrapped in a “black box”. One parameter set is defined as input; the model results are defined as output. Both deterministic and stochastic models can be analyzed.

SPOTPY scalability is realized by using the Python programming language, since it has an increasing support from the scientific community and is a recommended programming language for scientific research [32]. Pure Python code can run on every operating system without any complicated building mechanism. Parallel computing on HPC systems is supported by using a Message Passing Interface (MPI) code. Five of the eight implemented algorithms are suitable to for parallel computing (MC, LHS, SCE-UA, DE-MC_Z_, ROPE). The MPI code depends on the open source python package mpi4py [33]. A sequential run does not have any dependencies to non-standard python libraries.

SPOTPY is accessible as open-source on the Python package index PyPI and comes along with tutorials to allow a user-friendly start without the need of a graphical user interface and the benefit that everyone can use the most recent version of the code [34]. The code follows object-orientated style, where it supports modularity and is conform to the Open Source Definition [35].

### Structure of SPOTPY

**Fig 1:**
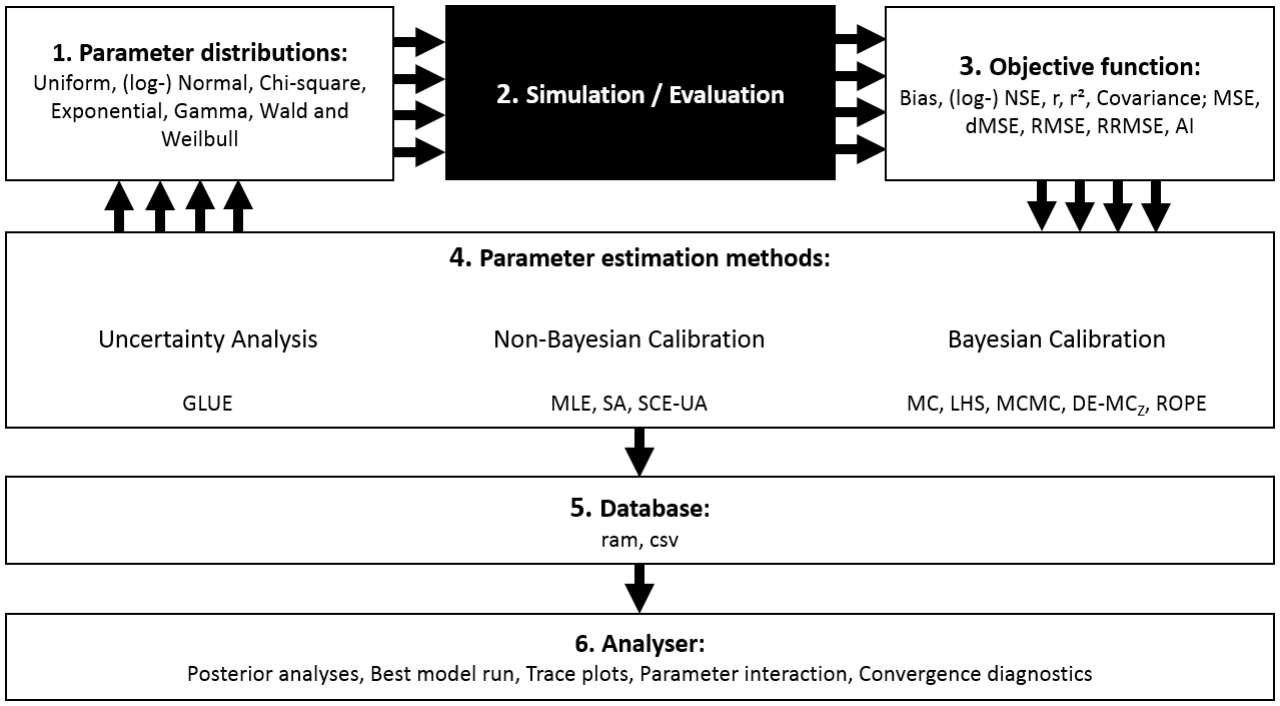
Flow diagram of the main processes captured with SPOTPY. Multiple cycle black arrows indicate the possibility of parallelization of the iterating algorithms. The black box returning the simulation and evaluation data can be filled with any model.

The design of SPOTPY brings different parameter estimation approaches within one set-up to allow users testing a variety of different combinations and methods. Fig 1 shows the main processes of this package, consisting of six consecutive steps when applying SPOTPY.

The different steps included are the following:

#### Step 1) Parameter distribution

Let *θ* = {*p*_1_, *p*_2_,…*p*_n_} be the initial input set of parameters of a (ecological-) model *M*. The ${\left\{p_{i}\right\}}_{i=1}^{n}$ random variables are selected from a joint probability prior distribution. This can be any user-defined distribution. We have pre-built the distributions Uniform, (log-) Normal, Chi-square, Exponential, Gamma, Wald and Weilbull with NumPy [36]. Each parameter *p*_*i*_ is marked with a user defined name, step size and optimal guess (initial parameter set), which are used as prior information by the algorithms and the database. The parameter name is used by the database, while the step size is an information needed for MCMC, MLE and SA to jump to the next point of the prior distribution. The optimal guess is the start point for all algorithms. The better this value is chosen, the faster convergence can be achieved.

#### Step 2) Simulation and evaluation

The output of *M* given a parameter set *θ*_*i*_ is defined as simulation *S*. The observed data *X* is characterized as evaluation. The simulation function is designed to call a model, returning a list of simulated values. The observation data is loaded in the evaluation function. One can also analyze a model with SPOTPY, which is only returning an objective function. Both functions and the following objective function offer the user flexibility to analyze almost every model with SPOTPY.

#### Step 3) Objective function

The objective function (also known as cost-function or goodness-of-fit-measure) quantifies how well the simulated data fits the evaluation data. Various objective functions are available (e.g. [37,38]) and have been proposed to account different sorts of errors in the simulation [39,40]. A guidance, which objective function to take under specific circumstances, is given by [41]. Hence, SPOTPY comes along with a wide set of objective functions, from which the user can select one or more for a specific issue (BIAS; Nash-Sutcliff efficiency (NSE); logarithmized Nash-Sutcliff efficiency (logNSE); Correlation Coefficient (r); Coefficient of Determination (r^2^); Covariance (cov); Decomposed (dRMSE), Relative (rRMSE) and Root Mean Squared Error (RMSE); Mean Absolute Error (MAE); Wilmott Agreement Index (AI)). The user has the option to combine different objective functions as only one function can be inaccurate [42]. A detailed description of the objective functions implemented in SPOTPY can be found for example in [43].

#### Step 4) Parameter estimation methods

The algorithms included in SPOTPY cover widely used parameter estimation methods from different approaches in recent publications. They can be connected with setup files containing the above-mentioned information about parameter distribution, simulation- and evaluation data as well as the objective functions. The simplest automatic parameter estimation method included is the MC method. It is used to sample random parameter values from a prior distribution. The structural LHS algorithm subdivides the distribution of each parameter into *m* equally probable non-overlapping intervals and creates a matrix by sampling from all created intervals. The algorithm has shown good projection properties [44–47]. MC and LHS can form the basis for the GLUE method [10,25], to get information about the posterior distribution of input parameters. GLUE has been widely applied in hydrology, but also in many other ecological disciplines, such as biogeochemistry or crop growth modeling [48–50]. If one is just interested in a fast calibration of a simple model (with nearly monotonically response function), the MLE is an efficient choice. To test whether the MLE algorithm is applicable for calibrating the desired model, it is recommend to test the model with MC first [51]. MLE maximizes the likelihood during the sampling, by adapting the parameter only in directions with an increasing likelihood. The famous Metropolis MCMC method can also deal with non-monotonically response functions. Nevertheless, it works similar as MLE. After each sampling, the likelihood is compared with last one. If the likelihood is better, the sampler jumps to the new sampled point. If not, it samples from the old position. Depending on a Metropolis decision, the sampler can also accept worse likelihoods (in order to avoid trapping at local optima). The MCMC algorithm can find a (quasi-) global optimum, but with a still remaining risk to stuck in local minima. The risk can be reduced by starting several chains/complexes that evolve individually in the parameter space. This technique is used in the global optimization strategy SCE-UA [1]. Each complex evolves independently to optimize the parameter. The population is periodically shuffled and new complexes are created with information from the previous complex. SCE-UA has found to be very robust in finding the global optimum of hydrological models and is one the most widely used algorithm in hydrological applications today [44]. Another robust method is SA. Thyer et al. [52] reported SA to be not as robust as the SCE-UA algorithm, but SA can be very efficient, when it is adopted to an optimization problem. After each step, a better objective function results in a new position. A worse objective function can be accepted with a Boltzman decision. If the new point is not accepted, the sampler jumps to a new parameter value. A variable controls a decreasing possibility to accept worse objective functions with increasing iterations. Thus, the risk to jump away from a global optimum is reduced. One of the most recent algorithms we present here is the DE-MC_Z_. It requires a minimal number of three chains that learn from each other during the sampling. It has the same Metropolis decision as the MCMC algorithm and has found to be quite efficient compared with other MCMC techniques [53]. Like SCE-UA and SA, DE-MC_Z_ does not require any prior distribution information. Another non-Bayesian approach is to determine parameter uncertainty estimations with the concept of data depth. This has the benefit, that the resulting parameter sets have proven to be more likely giving good results when space or time period of the model changes, e.g. for validation [54]. This approach is realized in the ROPE algorithm.

#### Step 5) Database

The database can store results from every parameter estimation method. Either in the working storage, which is fast, or in a csv file, which is comfortable. Saved information for every iteration are the objective function (-s), every parameter setting, optional the simulation results and the chain number (for algorithms with multiple threads like SCE-UA and DE-MC_Z_). The database can be analysed in any statistical software, programming language or the SPOTPY extension Analyser.

#### Step 6) Analyser

The Analyser module is an optional, but very powerful extension, which can read the SPOTPY database. Prebuild plots are provided for objective function and parameter traces, parameter interactions and best model runs. Posterior parameter sets can be selected and basic statistical analysis of the samples can be performed with Gelman-Rubin diagnostic [30] or the Geweke test [31].

To install SPOTPY, one just has to type *pip install spotpy* into the OS console. After that, SPOTPY can be used from any Python console:

import spotpy                 #Import the package

from spotpy_setup_rosenbrock import spotpy_setup #Import an example setup

sampler = spotpy.algorithms.sceua(model_setup()) #Initialize an algorithm

sampler.sample(10000)            #Run the model 10000 times

results = sampler.getdata()           #Load the results

from spotpy import analyser          #Import optional extension

spotpy.analyser.plot_parametertrace(results)    #Plot the results

### Set up of algorithms

The setting of the algorithms for the following case studies are depicted in Table 1. Two things are changed during the case studies: 1) The number of repetitions. 2) For efficiency reasons the set-up of the algorithms was slightly changed when, sampling from the Ackley function in the third case study: SA with Tini = 30, Ntemp = 30, SCE-UA with ngs = 2 and DE-MC_Z_ with nChains = dim.

**Table 1:** Settings of the algorithms used in the case studies.

Algorithm	Setting			Source
	Description	Abbreviation	Value	
**MC**	Normal random sampling			
**LHS**	Normal sampling along the HyperCube matrix			[3]
**MLE**	Percentage of repetitions dedicated as initial samples	burn-in	10%	
**MCMC**	Percentage of repetitions dedicated as initial samples	burn-in	10%	[4]
**SCE-UA**	Number of parameters	dim		[1]
	Number of complexes	ngs	2(dim)	
	Maximum number of evolution loops before convergence	kstop	50	
	The percentage change allowed in kstop loops before convergence	pcento	10^−5^	
	Convergence criterion	peps	10^−4^	
**SA**	Starting temperature	Tini	10	[6]
	Number of trials per temperature	Ntemp	10	
	Temperature reduction	alpha	0.99	
**DE-MC**_**Z**_	Number of different chains to employ	nChains	2(dim)	[2]
	Number of pairs of chains to base movements	DEpairs	2	
	Interval to save status	thin	1	
	Factor to jitter the chains	eps	0.04	
	Convergence criterion		0.9	
	Automatic adaption		True	
**ROPE**	Number of optimization cycles	subsets	5	[5]
	Acceptance ratio	percentage	0.05	

All settings of the algorithms should be adjusted, when dealing with other optimization problems.

For further detailed description of the SPOTPY package and the presented case studies see the download page (https://pypi.python.org/pypi/spotpy/ and the online documentation http://www.uni-giessen.de/cms/faculties/f09/institutes/ilr/hydro/download/spotpy).

## Case Studies

We show five different case studies to depict the capability of the different algorithms integrated in SPOTPY under different parameter optimization problems. Three of these case studies cover classical numerical optimization problems with a known posterior target distribution, one a hydrological model simulating real-world measured soil moisture values and one a biogeochemistry model where we tested the influence of different objective functions.

### Rosenbrock function

**Fig 2:**
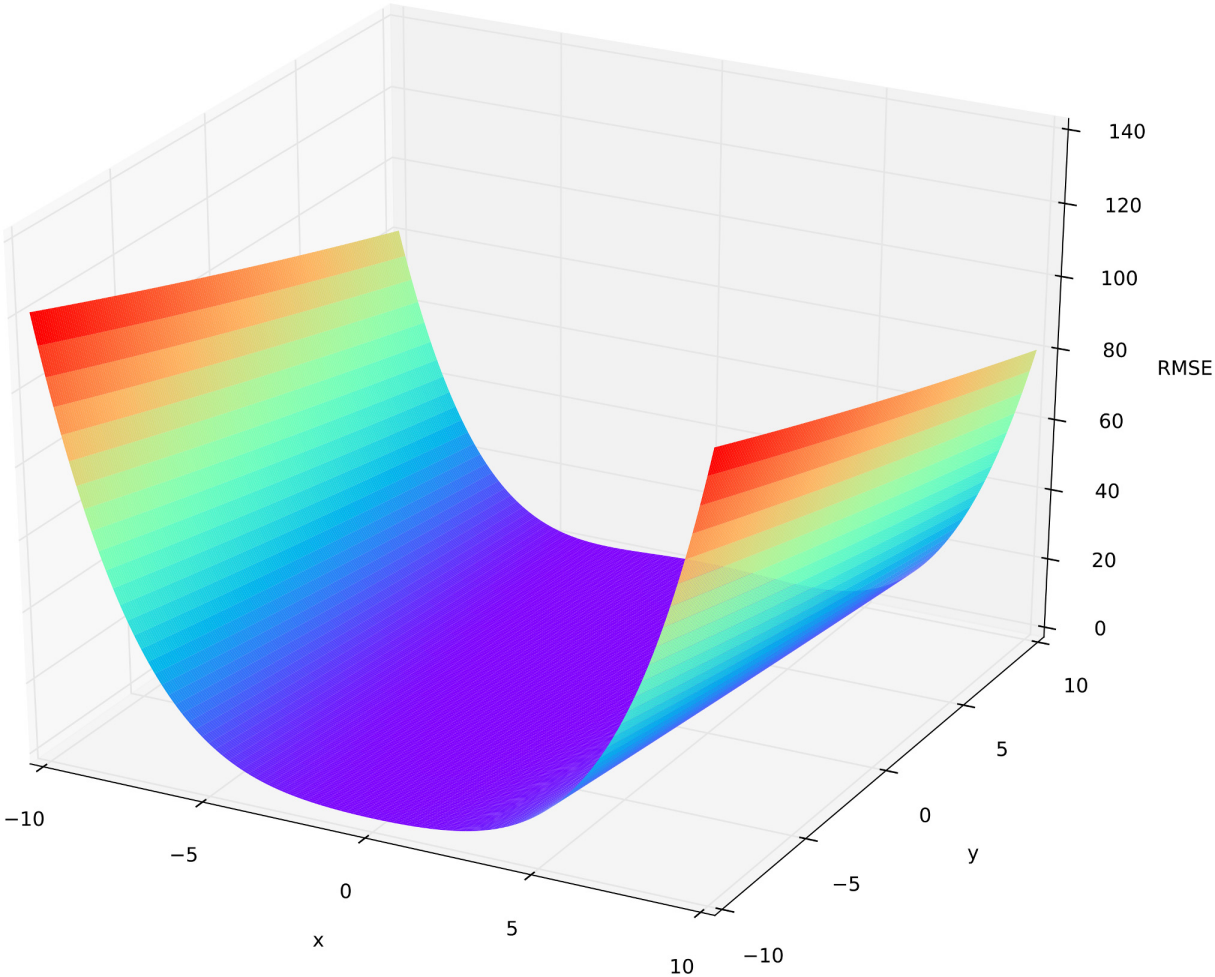
Three-dimensional surface plot of the Rosenbrock function. Colors from red (bad) to violet (optimal) represent the corresponding objective function (RMSE) for a parameter setting of x and y.

The Rosenbrock function [55] is often used to test and compare the performance of optimization methods [56–59]. It can be described as a flat parabolic valley (Fig 2) and is defined by

$$f_{Rosen}(x,y)={\left(1-x\right)}^{2}+100{\left(y-x^{2}\right)}^{2},$$ (1)

where we set the parameter space of the control variables to *x* ∈ [−10,10] and *y* ∈ [−10,10]. The global minimum is located at (*x*_*opt*_,*y*_*opt*_) = (1,1). At this point the function value is *f*_*Rosen*_(*x*,*y*) = 0. Due to its shape, it is an easy playground for optimization algorithms to find the flat valley, but it is hard to find the deepest point.

**Fig 3:**
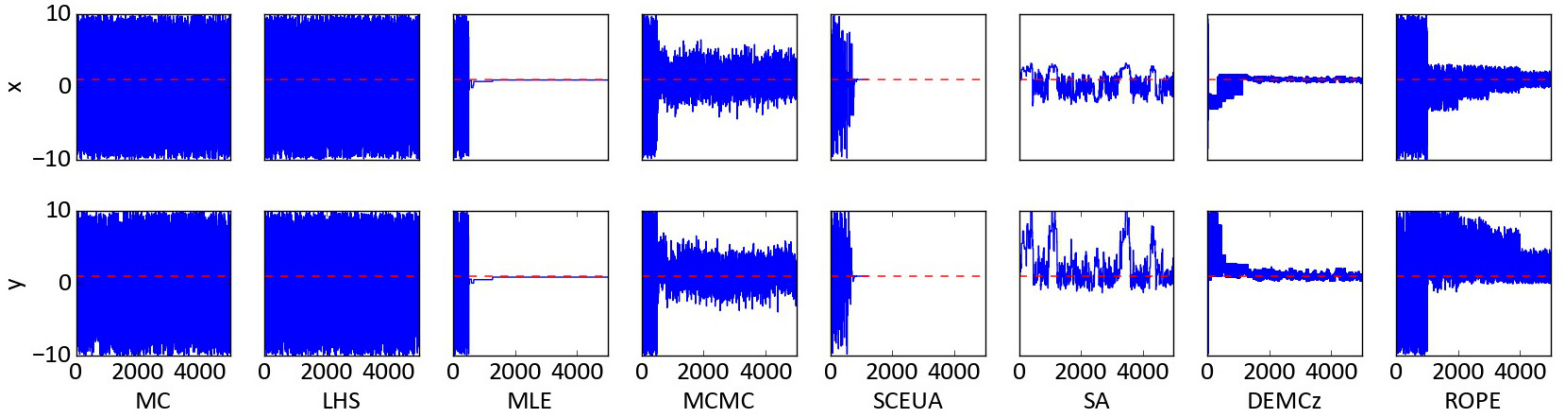
Trace plot of the two dimensional Rosenbrock function. The trace is shown as a blue line and the global optimum of the function as a broken red line. The x-axes show the number of iterations, while the y-axes show the value of the parameters x and y from -10 to 10.

Trace plots were created after sampling n = 5,000 times from parameter space of the Rosenbrock function. Fig 3 depicts the behavior of the algorithms. MC and LHS sample from the complete parameter distribution over the whole time. These algorithms find a few parameter distributions around the global optimum, which are masked by the overall large spread of selected parameter sets. All other algorithms show improved performances with increasing iterations. After 500 runs of burn-in, the MLE algorithm is very fast in finding the region around the global optimum. The MCMC works similar to the MLE, but with the possibility to jump away from the optimum. The algorithm finds the global optimum after 800 iterations and remains with a relative high uncertainty of x = 4 and y = 4. SA is fast in finding the valley and returns samples with a smaller uncertainty than MCMC. SCE-UA and DE-MC_Z_ sample in the first iterations over the whole range and converge at the global optimum after 800 and 1,000 iterations, respectively. SCE-UA stops after finding the exact global optimum. DE-MC_Z_ continues to produce parameter combinations close to the optimum with *x* ∈ [−0.5,0.5] and *y* ∈ [−0.5,1]. ROPE converges systematic closer to the optimum. The *y* variable range is reduced rather quickly to only positive values. For the *x* variable range the convergence works overall better. Overall, it turns out that MLE, MCMC, SCE-UA and DE-MC_Z_ are the most suited algorithms in finding the global optimum of the Rosenbrock function.

### Griewank function

**Fig 4:**
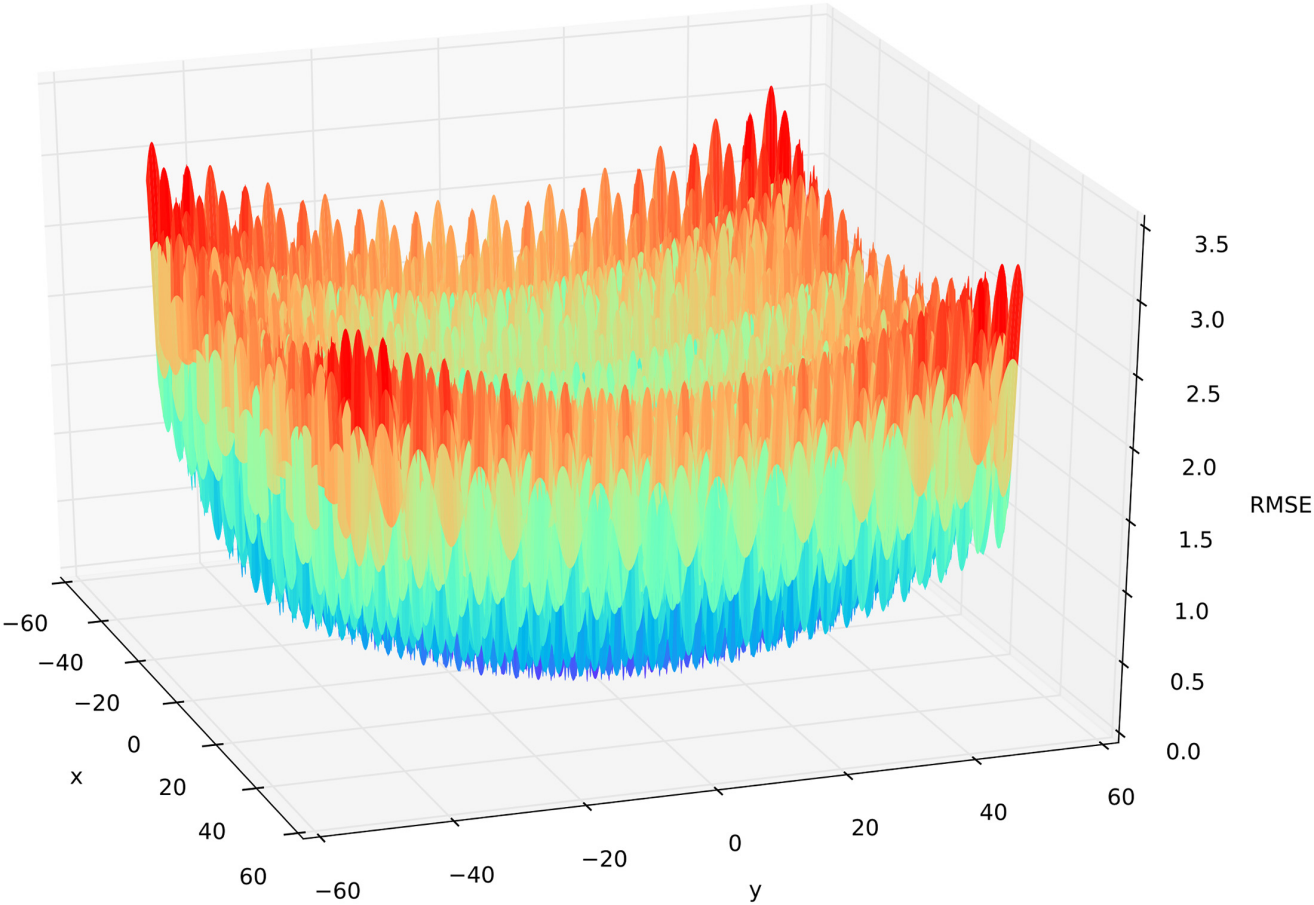
Three-dimensional surface plot of the Griewank function. Colors from red (bad) to violet (optimal) represent the corresponding objective function (RMSE) for a parameter setting of x and y.

The two dimensional Griewank function [60] is defined as

$$f_{Griewank}(x,y)=\frac{x^{2}+y^{2}}{4000}-\text{cos}\left(\frac{x}{\sqrt{2}}\right)\text{cos}\left(\frac{y}{\sqrt{3}}\right)+1,$$ (2)

where we selected the parameter space for *x* ∈ [−50,50] and *y* ∈ [−50,50]. One of the characteristics of the function is that it has many regularly distributed local minima (Fig 4), which makes it challenging to find the global optimum located at (*x*_*opt*_,*y*_*opt*_) = (0,0). The demanding function has been used for algorithm performance testing by others [61–63]. The surface of this function allows the investigation the algorithm performance under equifinality.

**Fig 5:**
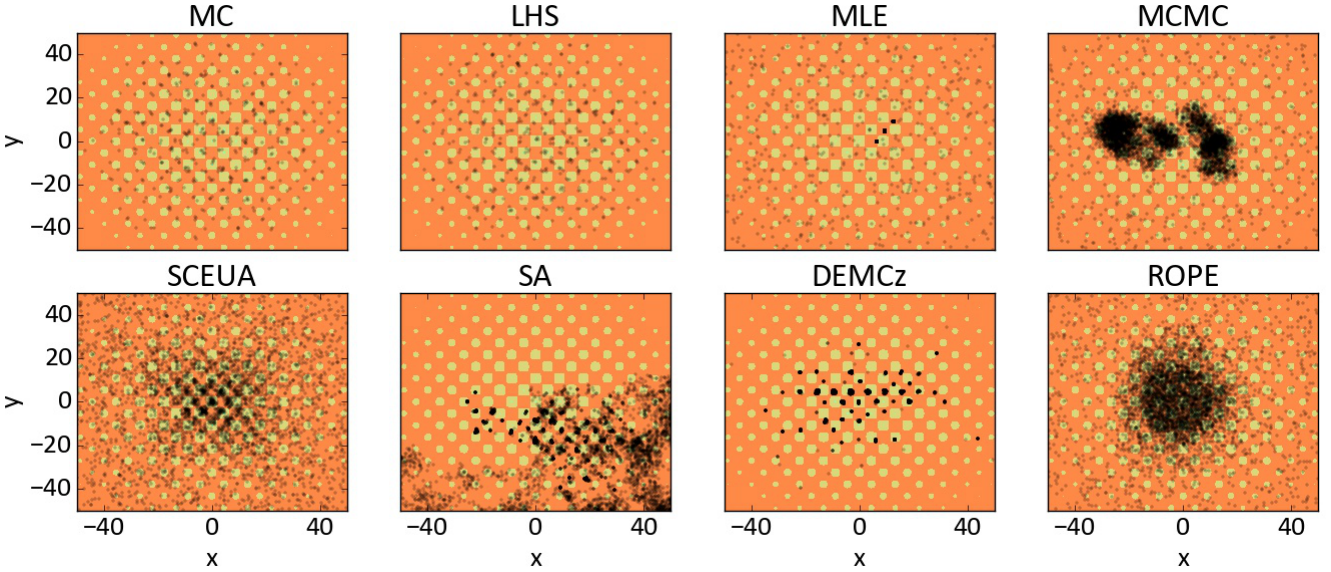
Surface plot of the Griewank function. Background colours showed from orange (bad response) to yellow (optimal response). Black dots show the sampled 5,000 parameter combinations. The x-axis shows the range of parameter x and the y-axis of parameter y.

The different algorithms were applied to the Griewank function (n = 5,000 iterations). The parameter interactions are shown as combined dotty plots (Fig 5). We added a surface plot of the Griewank function to show the locations of the various local minima. We conducted the GLUE methodology to MC and LHS by selecting the 10% best runs. One can see samples for MC and LHS on almost every local minima and the global optimum. The random walk of the MLE jumps between three local minima after the burn in, without finding the global optimum. The MCMC algorithm reaches several local minima in intermediate steps and found the global minimum. Nevertheless, the samples orientate not on the local minima and form clouds around the optimum. The SCE-UA samples parameter combinations from the whole range and reduces the range more and more to the global optimum. It stops the search after 4,000 iterations; nevertheless, the remaining parameter uncertainty is still high. SA did not find the optimal value and samples only negative values for the parameter y. DE-MC_Z_ found many local minima and the global optimum, which is representing the hilly response surface very good. ROPE reduced the investigated parameter range gradually centered to the optimal point.

### Ackley function

**Fig 6:**
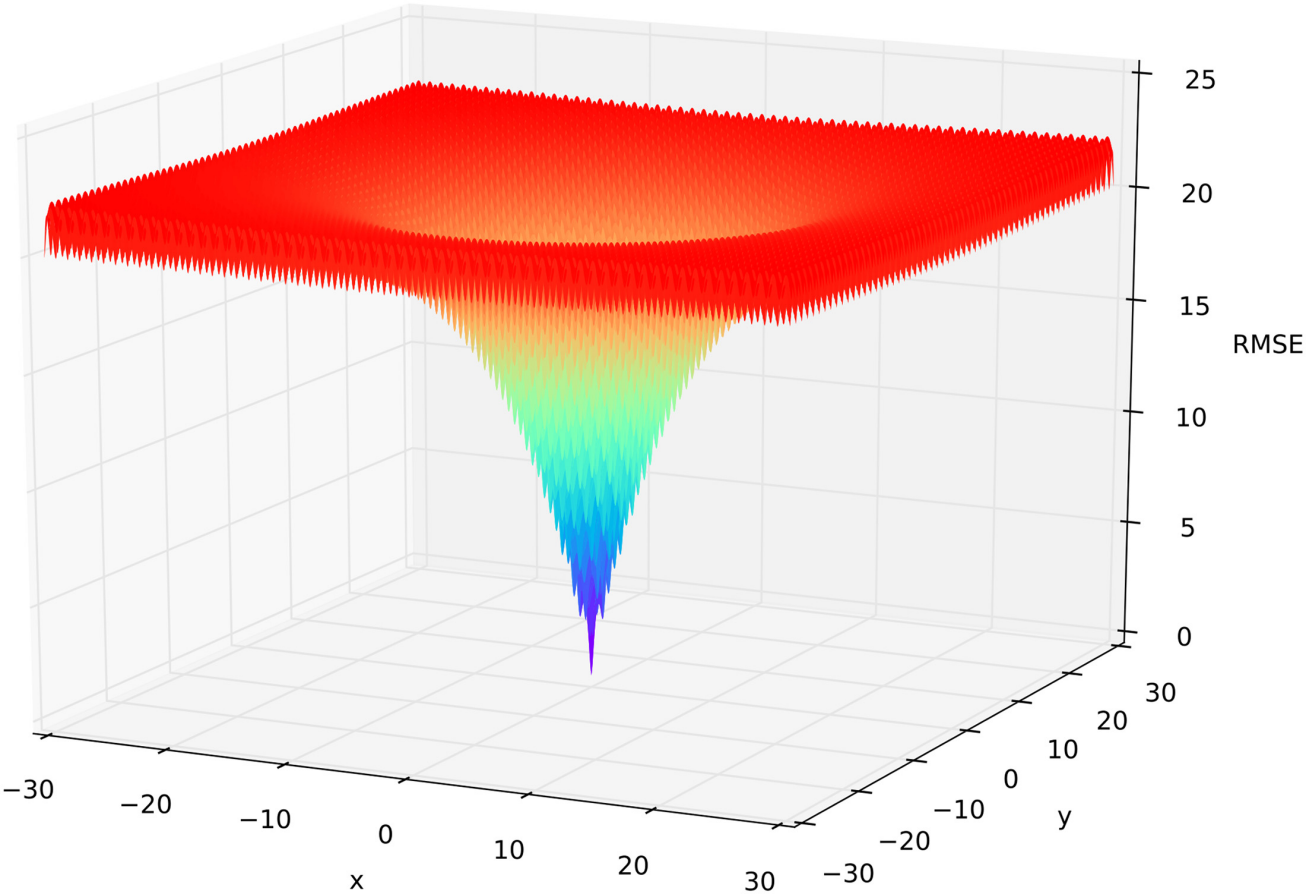
Three-dimensional surface plot of the Ackley function. Colors from red (bad) to violet (optimal) represent the corresponding objective function (RMSE) for a parameter setting of x and y.

The Ackley function is defined as

$$f_{Ackley}(x)=-20\text{exp}(-0.2\sqrt{\frac{1}{d}\sum_{i=1}^{d}x_{i}^{2}})-\text{exp}(\frac{1}{d}\sum_{i=1}^{d}\text{cos}(2\tau \tau x_{i}))+20+\text{exp}(1),$$ (3)

where **x** = (*x*_*i*_,..,*x*_*d*_) and the domain is defined as *x*_*i*_ ∈ [−32.768,32.768] [64]. The function has many regularly distributed local minima in the outer region, and a large funnel as the global optimum in the center located at *f*_*Ackley*_(0,…,0) = 0 (Fig 6). The function is widely used for algorithm testing [65–67]. We used setups with 2, 3, 5, 10, 20, 30 and 50 domains to investigate the algorithms behavior when dealing with an increasing number of parameters, while finding a very small global optimum.

**Fig 7:**
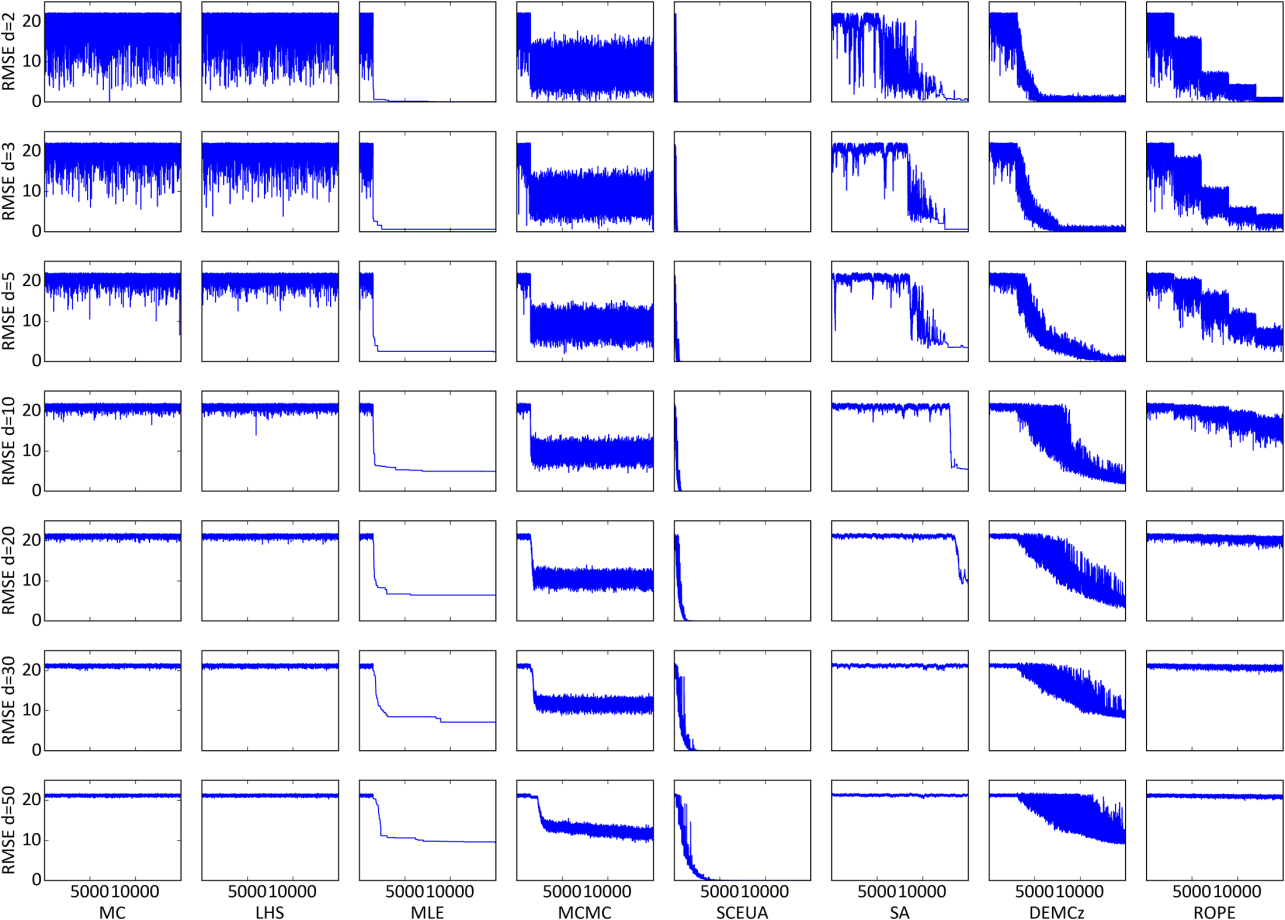
Objective function traces of the Ackley function. Setup with 2, 3, 5, 10, 20, 30 and 50 domains from the vector x of the Ackley function. All algorithms sampled 15,000 parameter combinations. The shown objective function on the y-axis is the root mean squared error (RMSE). The x-axis shows the number of iterations.

We used n = 15,000 iterations for every setup testing the algorithm’s performance (Fig 7). All algorithms perform worse with increasing dimensions. MC and LHS struggle even with two domains to find the exact optimum. With five domains, MLE, MCMC, SA and ROPE get close to the global optimum but do not find the exact position. With 10 domains DE-MC_Z_ does not reach the exact global optimum during the 15,000 iterations, but got close with a remaining RMSE of 2–5. With 20 and 30 domains MLE, MCMC and DE-MC_Z_ still give reasonable results, and can gather information during the iterations to get close to the optimum. Only SCE-UA is able to find the global optimum of the Ackley function with 50 domains during the given number of iterations.

### Catchment Modelling Framework

We used the Catchment Modelling Framework (CMF) developed by [68] to investigate the performance of the algorithms when dealing with a real measured world optimization problem. CMF is a toolbox to build water transport models from a set of pre-built process descriptions. The toolbox has been used before to model different catchments in one and two dimensions [45,49,69,70] and enables the test of hypotheses in hydrology [71]. In the application presented here, CMF is set up to simulate soil moisture in a one-dimensional soil column. Evapotranspiration is predicted by the Shuttleworth-Wallace method and soil water fluxes are modeled with the Richards equation. We searched for parameter sets to describe the shape of the water retention curve according to van Genuchten-Mualem [72] with four parameters: alpha, porosity, n and k_sat_. The prior parameter distributions are based on results from [49], where soil moisture was simulated with CMF for an agricultural site in Muencheberg. We used data from a Free Air Carbon dioxide Enrichment (FACE) grassland study site A1 in Linden, Germany [73]. The soil is classified as a Fluvic Gleysol. Meteorological data was used for the weather simulation and groundwater table data for the groundwater influence on this site. For the model evaluation, we utilized daily measured soil moisture data from the topsoil layer (0–0.1 m). The simulation time was from 01/06/1998 to 01/01/1999 as burn-in and simulation results until 01/01/2000 were used for evaluation.

**Fig 8:**
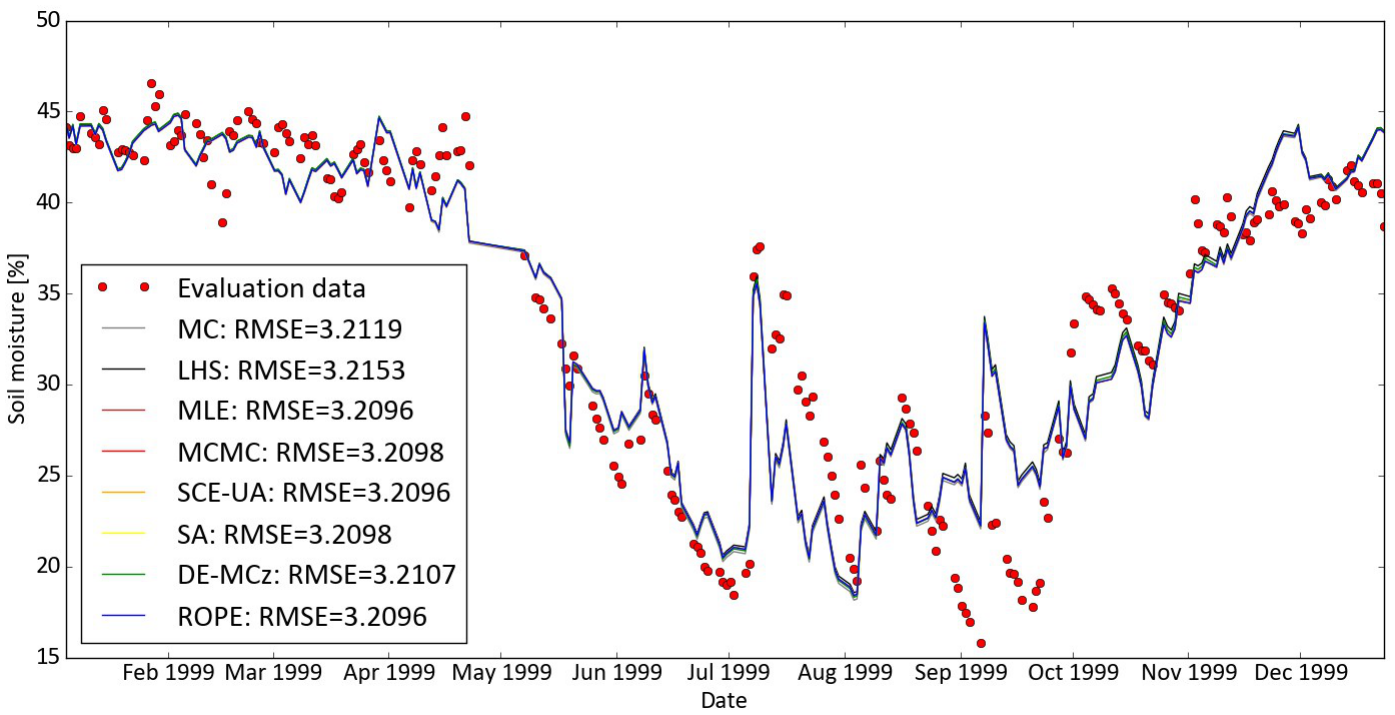
Best CMF runs for simulating soil moisture. Found with 10,000 iterations of the different algorithms realized with SPOTPY. The resulting different curves are very similar and overlap most of the time.

We started 10,000 iterations with a MPI structure. Twenty parallel threads on a HPC were used, resulting in a nearly linear speed up. The minimal RMSE was used to evaluate model performance. The best model runs of CMF found with the different algorithms are shown in Fig 8. All algorithms performed almost equally well. The ROPE, SCE-UA and MLE found the best parameter sets for predicting soil moisture with an RMSE as low as 3.2096. All other algorithms performed only slightly worse with RMSE between 3.2098 and 3.2153. Overall, the model simulations follow the main trend of the observations, especially during the first seven months when soil moisture decreased from 45 to 20%. The following flashy soil moisture curve is indicating that the model has deficiencies in simulating rapid changes in soil moisture of the uppermost soil layer, at least with the given forcing precipitation data and available information on soil parameters. This is a problem, which cannot be solved with parameter calibration and needs further investigation, e.g. by improving the model structure, adding more prior information into the process based model, or by testing other models.

**Fig 9:**
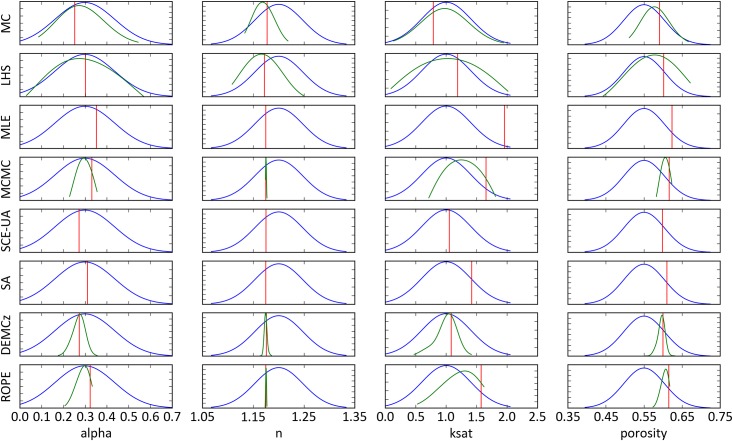
Prior distribution (blue line) of input parameters of CMF. Posterior distribution (green line) as the best 10% of the samples, plotted only for the Bayesian approaches. The optimal parameter setting is marked with a vertical red line.

Fig 9 shows the parameter distribution of the best performing parameter sets as well as the prior and posterior distribution (derived by selecting the best 10% of the sampling). The calibration algorithms MLE and SCE-UA resulted in a small posterior distribution. MCMC and DE-MC_Z_ reduced the parameter uncertainty of the posterior distribution by over 90% for parameter n and by 20% for parameter k_sat_. The other algorithms failed in reducing the parameter ranges. The optimal parameter setting for k_sat_ was found on a wide range from 0.8 (MLE) to 1.9 (MC) m day^-1^ and not in the center of the posterior distributions. Optimal settings for the parameter porosity were found in the upper range of the prior distribution, with small posterior distributions. The optimal parameter settings found for alpha, porosity and *n* are close to the center of the posterior distribution.

We do not know the true optimal parameter set of our hydrological model, or whether it exists at all. The optimal parameter sets we found differ from each other, indicating a high equifinality of the model. The optimal parameter settings for porosity were found in a small range from 0.6 to 0.63 for all algorithms. This values are in line with measured porosity of 0.60 to 0.65 [74]. The tested algorithms resulted all in similar best fits, with an RMSE = 3.2 Vol. % soil moisture. A direct comparison to other models is not possible, as this is the first study modelling soil moisture on the Linden FACE site. Nevertheless, results are not as good as others, e.g. [75] who used SCE-UA and found after 6,000 HYDRUS simulations remaining errors of RMSE = 0.03 Vol. % soil moisture on a different site. However, we attribute our relatively high remaining error to model deficiencies in capturing all natural effects, which might be a changing k_sat_ in the upper most soil layer after heavy rainfall on this site [76].

### LandscapeDNDC

**Fig 10:**
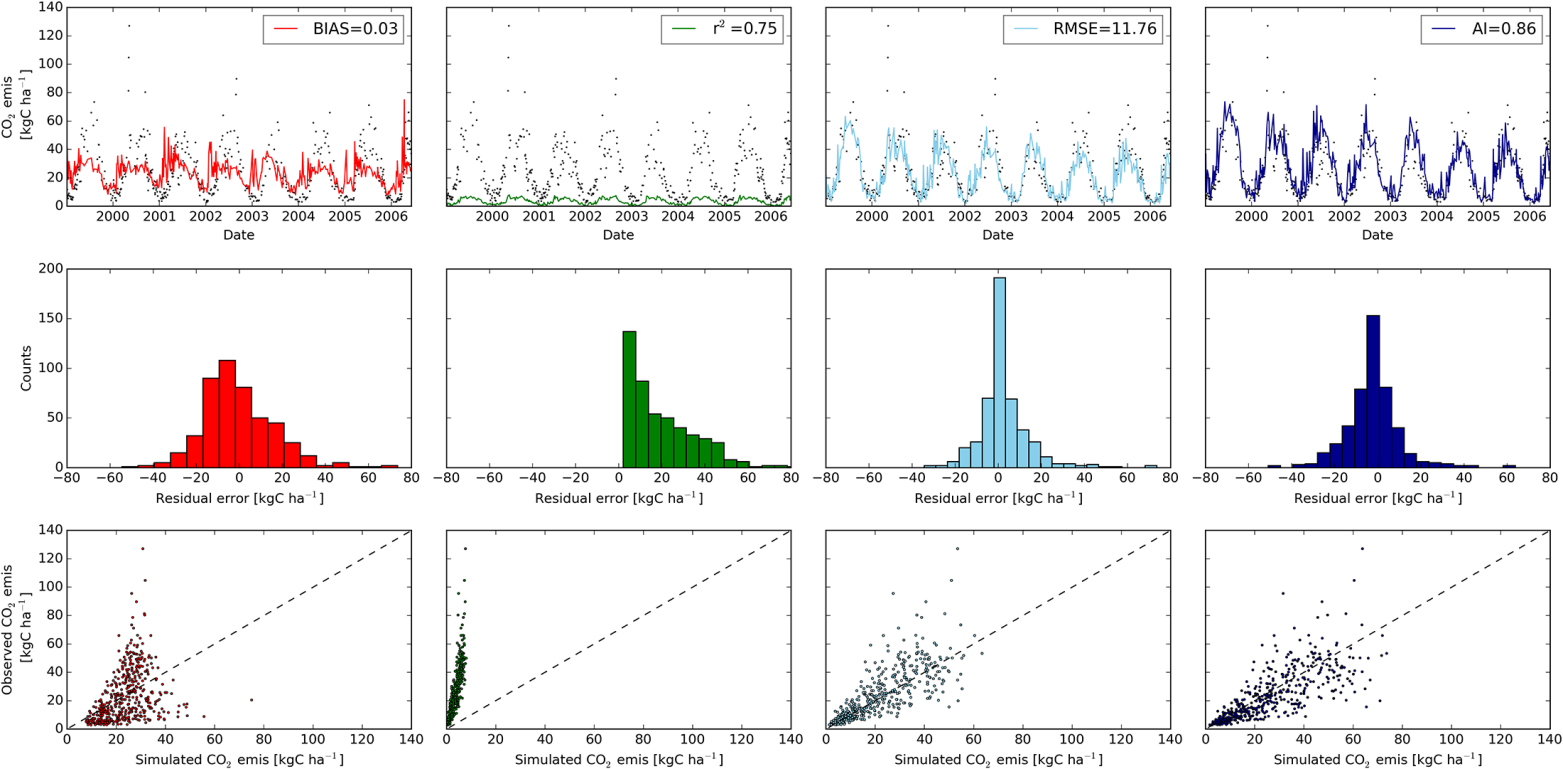
Comparison of measured and observed CO2 emission simulated with LDNDC (top panels). Best model runs were derived with four different objective functions using a Latin Hypercube sampling approach (n = 50,000 model urns). The objective function BIAS is shown in red, r^2^ in green, RMSE in light blue and AI in dark blue. Observed values are shown as black dots. Middle panels depict classified residual error counts of simulated CO_2_ emissions for each model. The dashed black lines in the correlation plots of observed versus simulated CO_2_ emissions (bottom panels) show the theoretical optimal fit.

We used LandscapeDNDC (LDNDC) developed by Haas et al. [69] to investigate the influence of the chosen objective function on the best selected model run. LDNDC is a biogeochemistry model to simulate greenhouse gas emissions and nutrient turn over processes. We used the model to simulate CO_2_ emissions from the soil of the Linden FACE site. The emissions were measured with the closed chamber method [74]. We setup the model with a warm-up period of one year and simulated the time from 01/01/1999 to 13/06/2006. Thirty parameters were sampled in a LHS with 50,000 runs. We selected four different widely used objective functions from SPOTPY to quantify the fit of the resulting simulations to the observations (Fig 10). The selected objective functions were the BIAS (ranging from -∞ to +∞, with 0 indicating an unbiased simulation), coefficient of determination (r^2^ ranging from 0 total disagreement, to 1 perfect regression), Root Mean Squared Error (RMSE ranging from -∞ total disagreement to 0 perfect fit) and the Wilmott Agreement Index (AI, ranging from 0 total disagreement to 1 perfect fit). The best BIAS found has a value of 0.03, which is close to its optimum of zero. However, soil emissions are overestimated in winter with 20 kg C ha^-1^ and underestimated in the summer months with 20 kg C ha^-1^. Looking at the distribution of the residuals, over- and underestimations are nearly Gaussian, resulting in a mean error near zero over the whole simulation period. The simulation with the best r^2^ has a relative high value of 0.75, but the simulations substantially underestimate the emissions from the soil during the whole model run. Nevertheless, the simulations follow the seasonal trend well, reflecting a reasonable timing of the model (Fig 10). To improve the fit of absolute emissions with the model, RMSE and AI are good options in SPOTPY. The distribution of the residual errors RMSE are narrower than the ones for AI, which indicates that the observations are better represented by the RMSE optimized model. In contrast, AI optimized simulations are superior in matching the absolute peaks of observed emissions.

## Discussion

All algorithms work well in SPOTPY, which was shown by the different case studies. Our intention was not to accept or reject algorithms but rather show their functionality within SPOTPY. Our results show reasonable effects, which have been reported in other algorithm comparison papers. The Rosenbrock case study showed us well performing algorithms when searching for a single optimal parameter set, like MLE and SCE-UA. Vrugt et al. [42] tested SCEM-UA (similar to SCE-UA) and MCMC on the Rosenbrock function, and reported that the first algorithm was faster in convergence. We found SA struggling in finding the optimum of the Rosenbrock, an observation also reported by Wang et al. [56]. When dealing with many local minima like it is true for the Griewank function, we got good results, when we conducted MC and LHS with the GLUE concept. They represent best the surface of the function. SCE-UA needed 4,000 iterations to stop the parameter search on the function, Jung et al. [28] found the optimum during 40,000 iterations. This difference in efficiency is most likely due to the setting of the algorithm. With an increasing amount of parameters on the Ackley function, we have seen good results for MLE, MCMC and DE-MC_Z_ and very good results for SCE-UA. Karaboga et al. [77] tested the swarm intelligence algorithm ABC on the Ackley function with 30 domains. They found after the optimum after 1,000 iterations, which is even better than the best performing algorithm of SPOTPY (SCE-UA). This algorithm could be a nice extension for the SPOTPY package. Behrangi et al. [78] used SCE-UA in a similar set up and found the optimum of a 30 dimensional Ackley function after around 4,000 iterations, exactly as we found it. Genetic algorithms give poor results on the Ackley function with 30 domains [77]. Madsen et al. [79] calibrated a hydrological model with SCE-UA and SA, showing that the first one worked better—similar to our case studies. Huang et al. [80] recommend MCMC to deal with many parameters. Our findings on the Ackley function show that evolution algorithms are even better suited for higher dimensional search problems. Ter Braak and Vrugt [2] showed that the evolution algorithm DE-MC_Z_ can be 5–26 times more efficient than MCMC. Gong [81] come to the same conclusion when testing the evolution algorithm SCEM-UA against the stochastic algorithm MLE. Good results were reported when using MC on a hydrological model with small parameter space [80]. We found that the rather simple MC and LHS often performed worse when searching the exact global optimum, but give reliable results under equifinality, like it is the case for our hydrological model build with CMF. We recommend using these simple search algorithms with the GLUE concept.

The LDNDC case study revealed that conclusions based on the model performance can be flawed when it is analysed with a not well-suited objective function. For example, the BIAS can reduce the overall model error, but it does not guarantee that the model fits the temporal variations of the observed data. The r^2^ is suited to find good parameter sets to predict timing of the system, but this objective function does not take the absolute values into account. RMSE and AI are well suited to find model realizations fitting the absolute values of the observed data. Legates and McCabe [40] pointed out that the coefficient of determination (r^2^) is inappropriate for model quantification because it is oversensitive to high flow but insensitive to additive and proportional differences between model simulations and observations. They recommended RMSE as the model evaluation tools.

Guinot et al. [82] generally classified objective functions into two types: distance-based objective function (e.g. RMSE) and weak form-based objective function (e.g. BIAS and r^2^). They concluded that although the distance-based objective functions have the advantage to search an identifiable model-parameter set, they may cause the local extremes in the response surface and lead to mis-calibration i.e. being trapped around secondary optima. By contrast, the weak form-based objective functions are more monotone than the distance-based objective functions. Depending on the aim of the model approach, it can be beneficial to combine several objective functions to find reliable posterior simulations [49]. While this is not a surprising or new result, the advantage of SPOTPY is, that it facilitates an easy comparison of currently eleven objective functions in a pre- and post-processing mode.

In general, the findings reveal that not every algorithm is suited for every parameter search problem. Even more, every algorithm has its advantages and disadvantages. Therefore, the overview in Table 2 showing the main capabilities of the algorithms might help the end-user to select a suited and efficient algorithm, without the need to understand and test every possible optimization technique. The approximate Bayesian compute techniques MC and LHS are very well suited to calibrate the model on multiple outputs with different objective functions. Nevertheless, they are very inefficient in high parameter space, like shown in the Ackley case study. Contrasting, the Metropolis MCMC method can be very efficient. However, it has the disadvantage that it is not possible to be used in parallel computing systems. DE-MC_Z_ is suited to be used in parallel, but gets inefficient when too many chains need to converge. ROPE is fully parallelizable but the generation of the parameter space after each subset needs a long computation time. All implemented non-Bayesian techniques (MLE, SCE-UA and SA) search only for one optimal parameter set, which makes them in general more efficient than the Bayesian approaches, but the outcome is very dependent on the used objective function and the parameter space, which is why they have to be chosen carefully. Furthermore, SCE-UA and SA need a pre-testing of the algorithm settings. They should not be used, without an adaption to a specific parameter search problems. MLE can be used straightforward, but the user has a higher risk to get stuck in a local optima. Unfortunately, there is no perfect algorithm and no perfect objective function. It depends. In this regard, SPOTPY was developed to help users to find their specific optimal solution.

**Table 2:** Capabilities of the different algorithms implemented in SPOTPY.

	MC	LHS	MLE	MCMC	SCE-UA	SA	DE-MC_Z_	ROPE
**Suited to investigate parameter uncertainty**	✓	✓		✓			✓	✓
**Allows considering multiple objective functions**	✓	✓						
**Possible to test prior parameter distributions**	✓		(✓)^a^	(✓)^a^	(✓)^a^		(✓)^a^	(✓)^a^
**Default algorithm-settings are all-round suited**	✓	✓	✓	✓				✓
**Suited for parallel computing**	✓	✓			(✓)^b^		(✓)^b^	✓
**Algorithm learns during sampling**			✓	✓	✓	✓	✓	✓

Checked fields indicate positive answers, fields with brackets are partly positive.

^a^ Only true during warm-up/burn-in

^b^ Only true up to the number of used chains/complexes. They are separated on different CPU cores.

## Conclusion

As a final aspect, we want to check, if our five defined criteria are met by SPOTPY. We conclude that SPOTPY is a broad package, combining several optimization approaches. We hope that it is helpful to users, as no other parameter estimation package provides such a wide range of implemented techniques and is so easy to use. Optimization experts can still accessed and adopted the complexity of the algorithms. Modularity is given as the entire package is coded in Python. The independency of SPOTPY makes it applicable to every model; in contrast to other packages, e.g. the presented toolbox of the SWAT [20]. The scalability claim of SPOTPY is valid. The straightforward MPI support results in a nearly linear time boost when analyzing time-consuming model runs and is as easy as tipping: *parallel = ‘mpi’*. Finally, the open-source accessibility of SPOTPY makes it available for everyone to every field of science, where parameter optimization is useful. We will maintain the code at least for the next two years and expand the functionality systematically. For instance, the most recent version comes along with a sensitivity analysis algorithm (FAST) and more possibilities to structure the simulation data in the database. Finally yet importantly, we welcome new contributors to share their results or to provide new ideas for features.

## Supporting Information

S1 File Simulation results of the Rosenbrock function. Contains the objective function traces for the 5,000 iterations with MC, LHS, MLE, MCMC, SA, SCE-UA. DE-MC_Z_ and ROPE.
(ZIP)

S2 File Simulation results of the Griewank function. Contains the objective function traces for the 5,000 iterations with MC, LHS, MLE, MCMC, SA, SCE-UA. DE-MC_Z_ and ROPE.
(ZIP)

S3 File Simulation results of the Ackley function. Contains the objective function traces for the 15,000 iterations with MC, LHS, MLE, MCMC, SA, SCE-UA. DE-MC_Z_ and ROPE.
(ZIP)

S4 File Simulation results of the CMF model with MC, LHS and MLE. Contains the objective function value, the corresponding parameters and the soil moisture simulations for the 10,000 model iterations with the algorithms MC, LHS and MLE.
(ZIP)

S5 File Simulation results of the CMF model with MCMC, SCE-UA and SA. Contains the objective function value, the corresponding parameters and the soil moisture simulations for the 10,000 model iterations with the algorithms MCMC, SCE-UA and SA.
(ZIP)

S6 File Simulation results of the CMF model with DE-MC_Z_ and ROPE. Contains the objective function value, the corresponding parameters and the soil moisture simulations for the 10,000 model iterations with the algorithms DE-MC_Z_, and ROPE.
(ZIP)

S7 File Best model runs of the LDNDC model. Contains the different objective function values and the corresponding CO_2_ emissions simulations.
(TXT)
